# Technical–economic analysis to identify the acceptable maximum attenuation on PON FTTH lines for wholesale network operators

**DOI:** 10.1038/s41598-023-39445-3

**Published:** 2023-07-29

**Authors:** Claudio Mazzei, Matteo Crescitelli, Daniele Fioramanti, Alessandro Quagliarini, Andrea Reale, Francesca Brunetti

**Affiliations:** 1 Università degli Studi di Roma “Tor Vergata”, 1 Via del Politecnico, 00133 Rome, Italy; 2 Fastweb S.P.A., 23 Piazzale Luigi Sturzo, 00142 Rome, Italy; 3 Open Fiber S.P.A., 449 Via Laurentina, 00142 Rome, Italy

**Keywords:** Fibre optics and optical communications, Information technology

## Abstract

Telecommunications companies are constantly chasing continuous technological advances with their management methods of Operations & Maintenance (O&M) that still struggle to turn their eye toward innovation and simplification of processes. In a future that aims at fully fiber-optic networks, the objective of the research is to propose guidelines and provide data to support the Wholesale Operator for the definition of the acceptable attenuation threshold on the Passive Optical Network Fiber-To-The-Home (PON FTTH) infrastructure to determine the Service Level Agreements (SLA) to be contracted with Retail Operators. Following exceeding the threshold, the Retail Operator has the right to open a Trouble Ticket to request the resolution of the anomaly. Consequently, the definition of the attenuation threshold strongly affects the number of Trouble Tickets to be managed and the related costs the Wholesale Operator bears. This paper analyzes a specific case of the PON FTTH network of the Italian wholesale operator Open Fiber. The studied infrastructure, with a length of 11 km, allows a maximum attenuation of 37 dB without degrading the service. An economic model has been proposed to assess the cost impact of moving the attenuation threshold in the SLA.

## Introduction

In recent years, the world of telecommunications has seen the birth of new wholesale-only companies with the goal of providing a single national network to allow Over The Top (OTT) operators to broadcast their content. Many of these companies aim to create future-proof, fully fiber-optic infrastructures to base the subsequent technological evolutions that will allow them to scale the expected traffic volumes in the years to come. Indeed, a significant increase in the demand for bandwidth is expected in the coming years, which will lead the Information & Communications Technology (ICT) industry to need appropriate access infrastructures to transport content; the most advanced technologies for this purpose are certainly FTTH and 5G^1–4^. This increase is not new; data consumption has grown exponentially since the birth of the Internet and the World Wide Web (www). Day after day, multimedia applications require the transmission of more data; for example, the video field saw a substantial technological transition that led High Definition Television (HDTV Full HD) to give way to the more recent super HDTV 4 K, which in turn will soon be replaced by 8 K resolution. HDTV 4 K consumes a few megabits per second, and 8 K will consume up to a hundred megabits per second^5^.

Consequently, the goal of many telecom operators around the world is to balance short-term investments and long-term goals^6^, evaluating all the technical–economic aspects to determine what the benefits and disadvantages of both technologies and infrastructure management approaches are^7–9^. Developing an infrastructure from scratch and evaluating all management and maintenance aspects requires enormous capital and operational investments^10^. Careful assessments must be made on the design and creation process of network^11,12^, which will determine its future scalability and the contractual aspects towards suppliers and customers^7^.

In fact, together with the costs of creating the network, the costs due to O&M activities and the agreements established in the contracts with the Retail Operators (who will use the infrastructure of the Wholesale Operator) must be taken into consideration with extreme care. It is crucial to reduce these future costs to a minimum^13^. Although there are already excellent solutions for the minimization of Capital Expenses (CapEx) and Operating Expenses (OpEx) in the construction of the network and its maintenance^14–16^, the literature and research on the optimization of costs and support for the definition of Service Level Agreements/Operation Level Agreements (SLA/OLA) are almost absent^8,17^. While many articles on PON FTTH network architectures are available, there is not much documentation on related economics^18–20^. It is evident that a complete understanding of the economy that revolves around new telecommunications infrastructures is still beyond the reach of the current literature.

In the context of O&M costs and penalties deriving from contracts with customers, a significant revolution was represented by the new definition of the concept of degradation on the PON FTTH infrastructure compared to the approach taken for decades on network architectures in copper^21^. Optical signals are immune to electromagnetic interference; thus, individual fibers can be bundled together during installation. Fiber optic cable is much less susceptible to environmental factors than copper cable^22^ and is immune to temperature and electromagnetic fluctuations^23^. On copper infrastructures, a Trouble Ticket can be opened for degradation due to problems related to attenuation, frequency, and phase. In contrast, a PON FTTH city access network of small size and designed according to international standards^24–26^, such as that of Open Fiber^27,28^, allows considering negligible the effect of degradation due to disturbances of frequency and phase. It is enough to consider the signal attenuation issues^29,30^.

The research presented in this paper aims to propose guidelines and provide data to support the Wholesale Operator for the definition of the acceptable attenuation threshold on their PON FTTH infrastructure in order to determine the SLA to be contracted with its customers, the Retail Operators. Following exceeding this threshold, the Retail Operator has the right to open a Trouble Ticket^30^ to request the resolution of the anomaly. Regarding the troubleshooting process, wholesale operators can efficiently and accurately measure the attenuation on the PON FTTH access infrastructure through the OTDR and the OLT and ONT transmission equipment^31^. To allow Retail Operators to perform the appropriate checks on the line before opening a Trouble Ticket, the Wholesale Operator can provide tools to perform checks on the specific end-user line. These requests are handled by OSS/NMS systems and directed—via SNMP/CORBA protocols—to the hardware that provides the service for the circuit—therefore, the OLT and the ONT. The parameters that are returned to the retail operator include the operational status of the ONT, the attenuation, the date/time of the last connection, etc.^32^.

It is easy to understand how the definition of the attenuation threshold can strongly affect the number of Trouble Tickets to be managed and, therefore, the management costs of the Wholesale Operator as well as the Customer Experience of the End User^33,34^. Furthermore, this SLA is crucial for PON FTTH dark fiber services, as it is the only measurable and certifiable parameter that the Wholesale Operator can provide to the Retail Operator.

The analysis starts from the observation that in the system specifications of the ITU-T 984.2^25^, the maximum optical budget available for B + SFP and C + SFP modules, which are used in Open Fiber’s network, are respectively 28 dB and 32 dB; these values are used for network design as well as for adjusting SLAs between Wholesale and Retail operators. However, the optical budget considers an excess margin due to phenomena such as aging and temperature excursions of the optical fiber^35^. Still, it does not exclude a proper operation of the infrastructure for greater attenuations.

This research aims to analyze the behavior of Open Fiber's PON FTTH infrastructure for higher attenuation than the value declared by the ITU-T standard. The goal is to extend the maximum attenuation threshold acceptable to Open Fiber in its contracts by a few dB without compromising the quality of the service offered. The results demonstrate that the current technology used at Open Fiber for PON FTTH infrastructures can ensure the proper functioning of customer circuits for attenuation up to 37 dB^16^. Considering this new threshold could allow Open Fiber to agree on a very different SLA with Retail Operators and, consequently, significantly reduce the opening of Trouble Tickets. This would lead to significant efficiency in troubleshooting activities and in terms of time and costs for the involved actors.

The intention is to share the technical–economic analysis in this paper with other wholesale-only telecommunications companies worldwide to allow the appropriate evaluations during the contract definition phase with their customers and suppliers. The methodologies used for the research will be described in the following paragraphs. Specifically, the configuration of the equipment and technologies used in the test plant will be described in detail to reproduce the PON FTTH infrastructure used in the Open Fiber context as closely as possible. Tests were performed on the network configuration with a no-loaded and overloaded network to evaluate any differences in behavior by the infrastructure. After highlighting the limits of Open Fiber's PON FTTH infrastructure, the bases were laid for calculating management costs relating to the SLA in question.

## Methods

**Figure 1:**
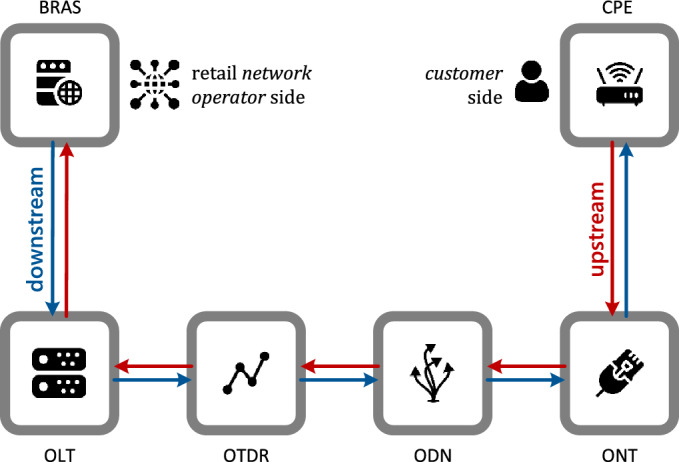
*Typical network setup*: typical network configuration used to provide the services contracted with customers.

**Figure 2:**
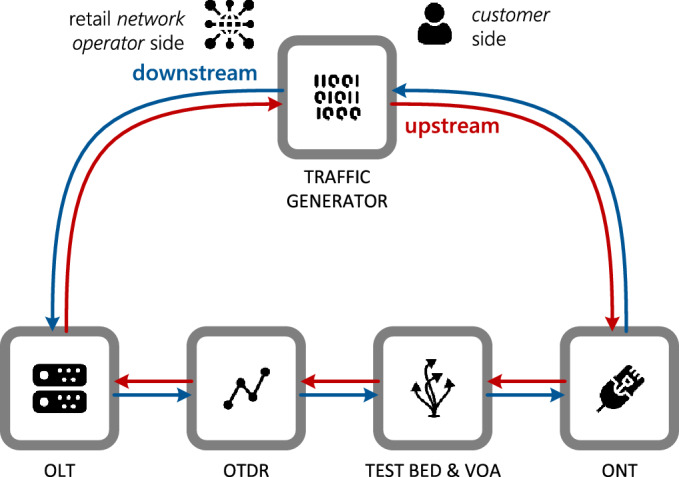
*Experimental Network Setup*: reproduction of the typical network configuration used to run the tests in the laboratory.

A setup reproducing the entire end-to-end (E2E) scenario—used to provide a service dedicated to a residential customer on PON FTTH infrastructure—has been realized in the test plant of the Open Fiber headquarters in Rome. Figure 1 briefly illustrates the network infrastructure with the Network Elements (NE) used to provide services for a residential customer (from now on, End User)^36^. At the same time, Fig. 2 shows the setup scheme reproduced in the Open Fiber test plant.

As regards Fig. 1, in the downstream direction, traffic transits from the Broadband Remote Access Server (BRAS) to the Customer-Premises Equipment (CPE) at the customer side; conversely, in the upstream direction, traffic transits from the CPE to the customer until it reaches the BRAS of the Network Operator. The access network—from the Point of Presence (PoP) to the customer side—consists of the following elements:

- Optical Line Termination (OLT) is a device that serves as the service provider endpoint of a passive optical network. It provides two main functions:
  1. To perform the conversion between the electrical signals used by the service provider's equipment and the fiber optic signals used by the passive optical network.
  2. To coordinate the multiplexing between the conversion devices on the other end of that network (called optical network terminals or optical network units).
- *Optical time-domain reflectometer (OTDR)* is an optoelectronic instrument to characterize an optical fiber. An OTDR is the optical equivalent of an electronic time-domain reflectometer. It injects a series of optical pulses into the fiber under test and extracts, from the same end of the fiber, light that is scattered (Rayleigh backscatter) or reflected from points along the fiber. The scattered or reflected light gathered back is used to characterize the optical fiber. Using the WDM technique, the test signal generated by the OTDR and the traffic signal share the same transmission medium. The OTDR test signal is indispensable for reflectometric analysis and knowing the quality parameters of the signals passing through the transmission medium.
- *Optical Distribution Network (ODN)* is a critical component of fiber-optic communication systems, specifically in the context of Passive Optical Networks (PONs). The ODN encompasses the physical infrastructure that extends from the central office or Point of Presence (POP) to end-user premises, facilitating the distribution of optical signals to multiple subscribers. In a PON architecture, the ODN is the backbone that connects the central office with individual users. It comprises various elements such as fiber optic cables, splitters, connectors, and closures. Fiber optic cables are responsible for transmitting optical signals over considerable distances, ensuring efficient and reliable data transmission^37^. Splitters are utilized to divide the optical signal into multiple paths, enabling simultaneous transmission to multiple subscribers without the need for active components in between. Connectors facilitate the seamless connection of fiber optic cables, ensuring optical continuity throughout the network. Conversely, closures provide protection and organization for fiber optic splices and connections, safeguarding them from environmental factors and ensuring proper maintenance.
- ONT (Optical Network Termination) is used on the customer side to terminate the fiber optical line (electric-to-optical signal processing) and demultiplex the signal into its data components (i.e., voice telephone, television, and Internet access). This is the last element of the optical network on the customer's premises. A Bragg reflector is applied to the ONT to reflect the OTDR wavelength.

As regards Fig. 2, new elements are introduced in the Experimental Network Setup, namely:

- Traffic Generator plays the role of CPE and BRAS by generating downstream and upstream traffic. Many of the measures used in this analysis were collected through its management system.
- Test Bed is the tool that reproduces the behavior of a classic ODN, the characteristics of which will be described later.
- Variable Optical Attenuator (VOA) is a device that can incrementally adjust the power of the optical signal passing through it. It is used to simulate disturbances in the infrastructure.

The Experimental Network Setup consists of the following hardware:

- Traffic generator: Ixia XGS12^38^;
- OLT: ZTE ZXA10 C600^39^; Alcatel Lucent Nokia 7360 ISAM FX-16^40^;
- ONT: ZTE ZXA10 F601^41^; Alcatel Lucent Nokia G-010G-R^42^;
- OTDR: Huawei N2510^43^;
- Optical fiber: ITU-T G.652 compliant^44^;
- OLT SFP modules: C + class^25^;
- ONT SFP modules: B + class^25^;
- Testbed: ODN length ~ 11 km.

Before starting the measurement activities, the instruments' and measurements' reliability was certified using multiple models and types of hardware indicated. Likewise, the Test Bed has been certified by Open Fiber with the support of its suppliers.

### Setup description and configuration

The *Traffic Generator* (*TG*) creates a stream of frames in continuous transmission mode with a length of 1526 Bytes. The connection between the TG and the OLT requires a 10 GE SFP module (1310 nm). After generation, the *Ethernet* (or *L2PDU*) *Frames* pass through the OLT, which radiates the ODN at a wavelength of 1490 nm.

In the upstream direction, the OLT aggregates the data flows from different PONs on a single flow. While in the downstream direction, the OLT obtains the traffic from BRAS and performs the reverse process—i.e., it turns the traffic towards the different ODN branches; in this experiment, TG plays the role of BRAS and CPE. As soon as it exits the OLT in the downstream direction, the carrier signal passes through the OTDR^45^, which couples the test signal to a second wavelength (1650 nm), which does not affect the carrier signal^46^. The OTDR is connected to the Test Bed using a Standard Connector/Angle Polished Connector (SC/APC) and to the OLT using a Lucent Connector/Angled Physical Contact (LC/APC). To accomplish its role, the OTDR employed for testing possesses multiplexing and demultiplexing capabilities. The multiplexer combines the OTDR's test signal with the live traffic transmitted over the network, ensuring integration into the overall signal mix. This combined signal is then distributed throughout the fiber network. At the receiving end, the demultiplexer separates the combined signals into their original components, including the OTDR test signal. Consequently, the OTDR receives and analyzes the reflected test signal along with other signals present in the fiber, supported by the demultiplexer's ability to extract wavelengths. As regards the analysis of the measures, the role of the OTDR was to certify the reliability of the physical attenuation found in the section between the OLT and the ONT.

Outgoing from the OTDR, the signal propagates through the TB, a scale reproduction of the ODN^47^. In this experiment, the network has an overall splitting ratio of 1:64. TB is composed as follows:

- The first level of splitting (1:4) is placed at a distance of 10 km; this distance was obtained using 4 × 2.5 km fiber spools.
- The second level of splitting (1:16); the four splitters are placed at a distance of 10.2 km, 10.4 km, 10.6 km, and 10.8 km.

TB simulates the PON and ensures the connection between one or more ONTs and the other devices (OTDR, OLT, etc.). Each ONT has an optical reflector, which allows the reflectometric analysis. The Bragg reflector reflects the test signal at 1650 nm to the OTDR and lets the traffic signal pass at 1490 nm. In this way, the connection quality to the End User is not affected. It's important to emphasize that in adherence to the standards, Open Fiber's network architecture consistently ensures that the distance between the first and last ONT never exceeds 20 km, and the splitting ratio is 1:64. These thresholds are consistently maintained, underscoring Open Fiber's commitment to compliance with established guidelines.

In a typical use at the Customer Site (as shown in Fig. 1), after the ONT—which delivers Ethernet Layer 2 (*Open Systems Interconnection—OSI model*) traffic—there is a further connection with a router that completes Layer 3 that is the Customer Premise Equipment (CPE). For the execution of the analysis, as already mentioned, the role of the CPE was played by the TG through the creation of a loop. In fact, in this case, Traffic Generator behaves like a CPE, as evident in Fig. 2.

The transport of the traffic of the Retail Operator within the network is managed using the 802.1Q protocol. IEEE 802.1Q—often referred to as Dot1q—is the networking standard that supports Virtual LANs (VLANs) on an IEEE 802.3 Ethernet network. The standard defines a system of VLAN tagging for Ethernet frames and the accompanying procedures to be used by bridges and switches in handling such frames^48^. Consequently, a VLAN had to be configured for each ONT to segregate broadcast domains. As for the traffic bandwidth, the network has been tested in discharged (with only one ONT) and charged (with three ONTs that saturate the PON) conditions. Standard SFP modules for Gigabit PON technology enable transmission speeds of 2.488 Gbps downstream and 1.244 Gbps upstream.

The network analysis has focused on Layer 2 (Data Link Layer) parameters of the ISO/OSI model^49^, specifically the Throughput and Frame Loss Ratio (hereafter referred to as FLR)^50^. Attenuation and Return Loss were induced by reproducing some real situations (such as the bending of the fiber and dirty connectors) to analyze and simulate the degradation phenomenon on the PON FTTH infrastructure^51^. The primary method that was applied involved using a VOA (airgap type)^52^, which allows for varying the Insertion Loss on the channel. The consequent passage of a smaller or larger quantity of air causes the insertion loss variation on the optical channel^53^. In addition, fixed attenuators can be combined with the VOA.

As for the induced bending, the fiber was placed on a PVC cylinder with a radius of curvature of 0.25 cm. To study the phenomenon, test measurements were collected by constantly increasing the windings of the fiber around the cylinder, up to a total of 8. Then, all measurements were collected, leaving the fiber wrapped in PVC with 8 windings^54^. The fiber was stressed for a total of two weeks with bending tests, while the connectors were soiled by tactile contact and oils.

The analysis provides the execution of two types of tests:

1. Test with no-loaded network using a single ONT—i.e., without saturating the downstream and the upstream channels.
2. Test with overloaded network using three ONTs—i.e., saturating both the downstream and upstream channels.

The starting attenuation included in the system was fixed at 30 dB, and subsequently, it was increased using the VOA. The effect of the attenuation introduced in the network has been observed by collecting the correlation between *Attenuation* [dB], *Throughput* [Mbps], and *FLR* [%]. It is essential to specify that the data shown in the graphs of this research refer only to the payloads of the data transmitted by the TG in both directions and that no FEC algorithms were applied. Since Open Fiber's role is to transport traffic up to Layer 2 of the ISO/OSI stack, the various types of traffic that can be transmitted by a Retail Operator on their network (e.g., IPTV, VoIP, etc.) have not been taken into consideration during the test. As a Wholesale Network Operator, Open Fiber’s main goal is to guarantee the maximum quality of service for the Retail Operators and their End Users despite the transmitted traffic data type.

## Results

### No-loaded network

The first experiment focused on the most basic situation, corresponding to having only one customer on the PON; this corresponds to having the total capacity available downstream and upstream. The ONT device under examination was placed at a distance of 10,697 m. TCONT Type 3 and Status Reporting Dynamic Bandwidth Allocation (SR DBA) algorithm were used for upstream transmission.

The following Traffic Bandwidth and Traffic Profile were configured for the test:

- Traffic Bandwidth:
  - Downstream—Peak Information Rate (PIR): 1 Gbps;
  - Upstream—PIR: 330 Mbps.
- Traffic Profile: MonoCoS (Class of Service). ^48,55^

**Figure 3:**
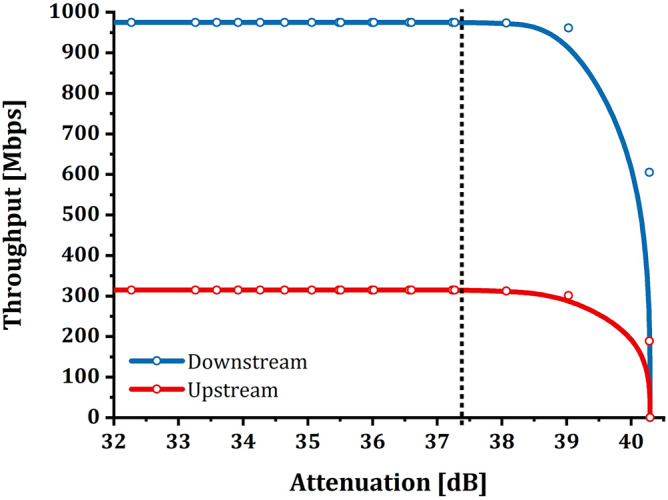
1 ONT setup—throughput/attenuation relation: the graph shows the relationship between throughput (Mbps) and attenuation (dB) for the setup consisting of only one ONT on the GPON. It is possible to note that throughput does not suffer from induced attenuation up to values of about 37.4 dB; after this value, the quantity of data successfully transmitted decreases considerably until a complete signal loss, coinciding with the loss of communication between OLT and ONT. The experimental samples were plotted using a B-Spline function as a guide for the eye.

Figure 3 graphs the throughput—i.e., the amount of data successfully transmitted in a unit of time, measured in the downstream direction (1490 nm) and in the upstream direction (1310 nm).

The upstream and downstream curves reported in Fig. 3 highlight three intervals of interest in which the behavior of the traffic passing through the link varies in the same way in both directions:

- The first range starts from 0 dB up to 37.4 dB. In this interval, it is evident that the behavior of the ONT does not show anomalies or even decreases in throughput, which remains constant and stable. Even the ONT under examination does not indicate problems through its LEDs; in fact, the one relating to the PON connection is green and solid, and the power level at the ONT port is around − 32.5 dBm. The C + /B + SFP modules’ thresholds mentioned during the introduction of this article have been substantially exceeded, but the circuit is still fully working, and no packet discards are found.
- The second range starts from 37.4 dB up to 40.3 dB. In this interval, a condition of signal degradation begins to occur, highlighted by the “descent” of the throughput curve. Hence, link degradation is causing information packets to be discarded. Although concentrated in a couple of dB, the "descent" is gradual and does not have an "on/off" behavior. The circuit is still working, but it is not guaranteeing the expected performance. The ONT shows a condition of the LEDs unchanged from the previous interval, and the power level at the ONT port is around − 35.2 dBm. Under these conditions, the customer typically encounters problems on the line, and the Retail Operator has the right, by contract, to open a Trouble Ticket to the Wholesale Operator to get the issue resolved.
- The third range is the one that goes from 40.3 dB onwards and represents the complete loss of the signal—i.e., the outage. There are no conditions to ensure proper communication between the OLT and the ONT. The ONT shows the LOS LED in red and fixed color.

**Figure 4:**
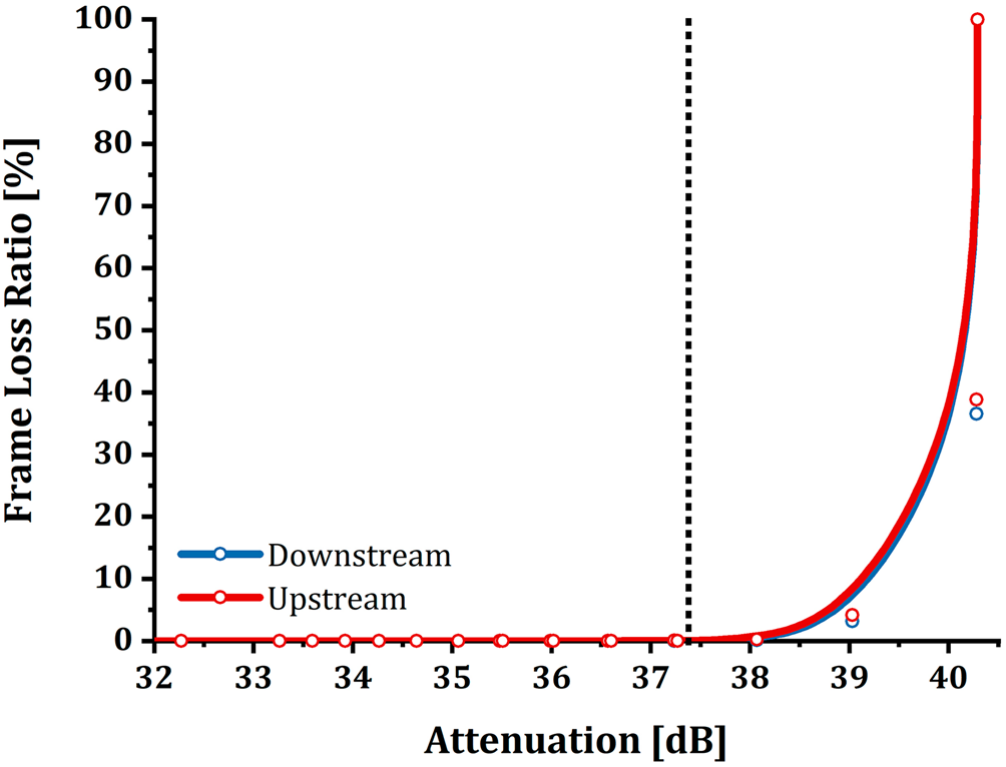
1 ONT setup—frame loss ratio/attenuation relation: the graphs show the relationship between FLR (%) and attenuation (dB) for the setup consisting of only one ONT active on the GPON. As described for throughput, it is possible to note that FLR does not suffer from induced attenuation up to values of about 37.4 dB; after this value, the percentage of data transmitted successfully decreases considerably until a complete loss of frames coincides with the loss of communication between OLT and ONT. The experimental samples were plotted using a B-Spline function as a guide for the eye.

Experimentally it has been observed that when reaching the 40.3 dB attenuation, it is necessary to reduce the attenuation by at least 3 dB to allow OLT and ONT to re-establish coherent communication. In this case, the LEDs of the devices do not show signs of anomalies even though the connection is still experiencing information discarding (as mentioned above for the second range). As anticipated, the analysis is deepened and enriched by showing the relationship between attenuation and FLR (Fig. 4). The graph in Fig. 4 was made by calculating the percentage values ​​of FLR as follows:

Downstream: the subtraction between the frames transmitted by the TG and those received by the ONT.

1 $${\text{FL}}{\text{R}}_{\text{ds}}=\frac{{\Delta }_{\text{ds}}}{{\text{TxFrames}}_{\text{TG}}}=\frac{{\text{TxFrames}}_{\text{TG}}-{\text{RxFrames}}_{\text{ONT}}}{{\text{TxFrames}}_{\text{TG}}}$$

Upstream: the subtraction between the frames (conveyed into bursts) transmitted by the ONT and the frames received by the TG.

2 $${\text{FL}}{\text{R}}_{\text{us}}=\frac{{\Delta }_{\text{us}}}{{\text{TxFrame}}{\text{s}}_{\text{ONT}}}=\frac{{\text{TxFrame}}{\text{s}}_{\text{ONT}}-{\text{RxFrame}}{\text{s}}_{\text{TG}}}{{\text{TxFrame}}{\text{s}}_{\text{ONT}}}$$

It is possible to notice how the FLR behavior (Fig. 4) traces the trend of the throughput as expected. The three intervals identified previously for the throughput are the same in terms of dependence on attenuation.

### Overloaded network

The behavior of the overloaded network has been tested using three ONTs with the same stress modes reported before. In this case, the use of three ONTs allows reaching the use of the maximum downstream channel capacity that is represented by 2488 Gbps. TCONT Type 3 and SR DBA were used for upstream transmission.

The following Traffic Bandwidth and Traffic Profile were configured for the test:

- #1, #2 ONT:
  - Traffic Bandwidth:
    - Downstream—Peak Information Rate (PIR): 1 Gbps;
    - Upstream—PIR: 330 Mbps.
  - Traffic Profile: MonoCoS (Class of Service).
- #3 ONT:
  - Traffic Bandwidth:
    - Downstream—Peak Information Rate (PIR): 500 Mpbs;
    - Upstream—PIR: 330 Mbps.
  - Traffic Profile: MonoCoS (Class of Service).

**Figure 5:**
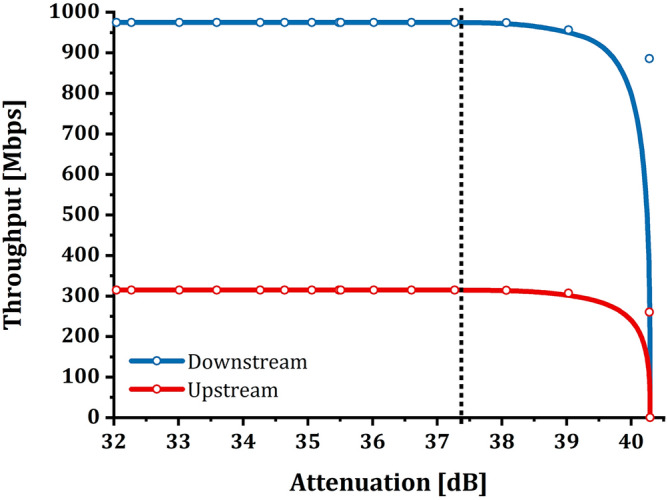
3 ONT setup—throughput/attenuation relation: the graphs show the relationship between throughput (Mbps) and attenuation (dB) for the setup consisting of three ONT on the PON. It is possible to note that throughput does not suffer from induced attenuation up to values of about 37.4 dB; after this value, the quantity of data successfully transmitted decreases considerably until the complete loss of the signal, coinciding with the loss of communication between OLT and ONT. The experimental samples were plotted using a B-Spline function as a guide for the eye. The chart shows the curves of only one ONT out of the three available, as the curves of the latter overlap entirely.

Figure 5 shows the relationship between attenuation and throughput, but this time the configuration foresees three connected devices that saturate the downstream and upstream channels. The values ​​refer to the same ONT analyzed previously and placed at 10,697 m from the OLT.

Compared to the previous case, the signal loss has no significant differences. The signal mirrors the three ranges described above for the first test with the no-loaded network. Hence, throughput is constant up to 37.4 dB, after which an increasing packet loss begins to occur up to 40.3 dB, at which time the signal is completely lost. From experimental evidence, it can be supposed that the contemporaneity of the transmission of several ONTs and the saturation of the channel on the PON FTTH infrastructure does not modify the behavior of the single circuit.

**Figure 6:**
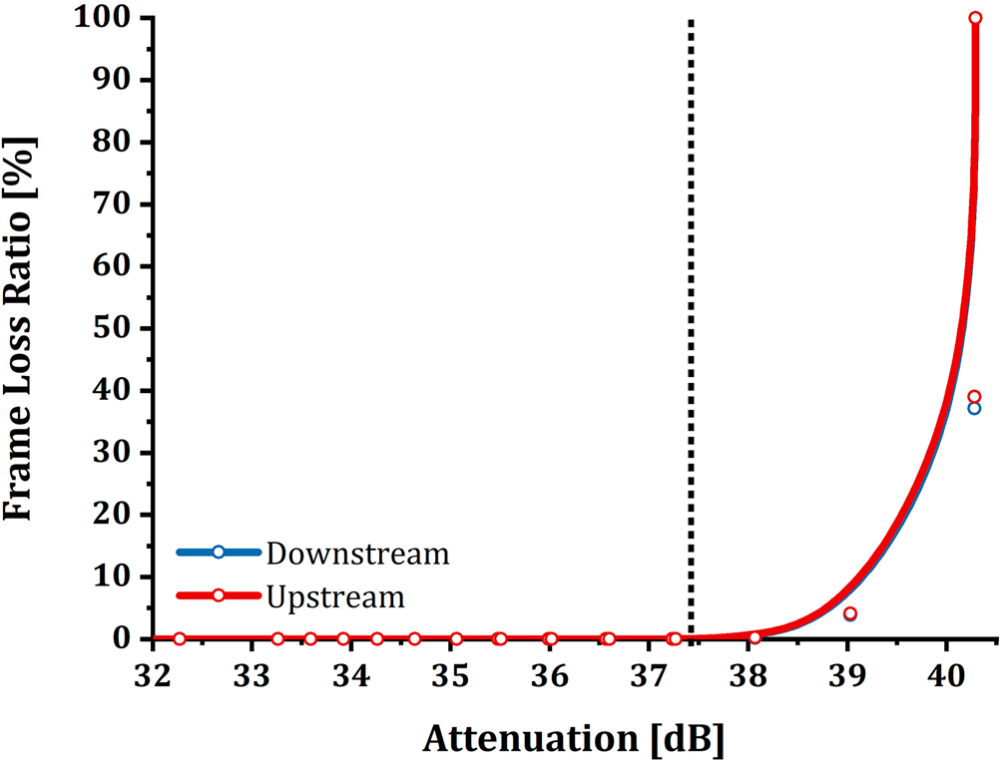
3 ONT setup—frame loss ratio/attenuation relation: the graphs show the relationship between FLR (%) and attenuation (dB) for the setup consisting of three ONT on the GPON. As described for throughput, it is possible to note that FLR does not suffer from induced attenuation up to values of about 37.4 dB; after this value, the percentage of data transmitted successfully decreases considerably until a complete loss of frames coincides with the loss of communication between OLT and ONT. The experimental samples were plotted using a B-Spline function as a guide for the eye. The chart shows the curves of only one ONT out of the three available, as the curves of the latter overlap entirely.

As it was easy to imagine, also about FLR (shown in Fig. 6), the behavior is the same as for the no-loaded network tests. As attenuation increases, there is always an increase in dropped packets.

### Technical–economic considerations

The results obtained in this research provide the tools for Wholesale Operators to accurately define the agreements in the contracts with their customers, Retail Operators, who will resell their service on PON FTTH infrastructure to end-users. Specifically, the SLA for which the research analysis was performed relates to the maximum acceptable attenuation on the PON FTTH infrastructure. As described in the introduction of this paper, this SLA assumes a fundamental character as, in the case of a Trouble Ticket, it is a parameter that can be certified by the Wholesale Operator through its test tools^56^.

The foundations for a cost model have been laid to permit the Wholesale Operator to assess the service levels to offer its customers. This model allows the Wholesale Operator to indicatively determine the cost it would incur by establishing a certain attenuation threshold. As described above, exceeding this threshold enables the Retail Operator to open a Trouble Ticket—i.e., reporting a problem to the Wholesale Operator, owner of the network infrastructure.

The management of the Trouble Ticket involves costs depending on specific conditions, such as the contract agreements that the Wholesale Operator has stipulated with its suppliers or the organizational processes within the company itself. Consequently, these factors may vary from one company to another. The Wholesale Operator intending to use this model may feel free to delete or add additional elements based on their business context and supplier relationship.

Before introducing the factors that can be part of the cost model mentioned above, the main actors involved in the management of a failure on a telecommunications network are reported:

- *NOC* (*Network Operations Center*) the operating unit (internal or external to the company) that acts as a point of contact between the Wholesale Operator and the Retail Operator and manages the entire life cycle of the Trouble Ticket, then:
  - Categorize and prioritize the Trouble Ticket.
  - Carry out an initial diagnosis of the problem reported in the Trouble Ticket.
  - If the problem cannot be solved remotely, notify the field technicians who will take care of reaching the point where the problem was located to solve it.
- *Field Technicians* the technicians (internal or external to the company) responsible for managing the resolution of the problem (Trouble Ticket) at the customer site or, more generally, at the place where the fault was located.
- *Contact Center* the staff (internal or external to the company) who are in charge of contacting the customer to arrange any appointments at his site.

Therefore, some of the factors that can be considered within the cost model and involve the actors mentioned above are listed below—these factors determine the costs that would result from choosing a specific attenuation threshold relating to the SLA mentioned earlier. Before going into the definition of the specific factors, here is a list of all the parameters that characterize them:

- $FTE$ = Full-Time Equivalent, the calculation of full-time equivalent (FTE) is an employee's scheduled hours divided by the employer's hours for a full-time workweek.
- $\#TT$ = is the annual quantity of Trouble Tickets to be considered; two fundamental assumptions are made on this parameter for the model to make sense:
  1. A Retail Operator can potentially open a Trouble Ticket for each of its customers, having a greater attenuation than the threshold set by the SLA.
  2. Only one Trouble Ticket can be considered for a specific customer during the year; therefore, it is assumed that any processing of the Trouble Ticket was decisive.
- ${{\EUR}}_{{{\text{Salary}}}}$ = is the average annual salary of an employee who holds a specific role.
- ${{\EUR}}_{{{\text{TT}}_{{{\text{NOC}}}} }}$ = is the unit cost for processing a Trouble Ticket by the NOC.
- ${{\EUR}}_{{{\text{TT}}_{{{\text{{{On - Field}}}}}} }}$ = is the unit cost for processing a Trouble Ticket by field technicians.
- ${{\EUR}}_{{{\text{Call}}}}$ = is the unit cost of a call to the customer by the Contact Center.
- ${{\EUR}}_{{{\text{Fine}}}}$ = is the unit cost of a fine to be paid to the Retail Operator.
- ${t}_{{TT}_{NOC}}$ = is the average resolution time (expressed in hours) of a Trouble Ticket by the NOC.
- ${t}_{{TT}_{Call}}$ = is the average call duration (expressed in hours) to the customer by the Contact Center.
- ${t}_{{TT}_{\text{On - Field}}}$ = is the average resolution time (expressed in hours) of a Trouble Ticket by a field technician.
- ${{\%}}_{\text{On - Field}}$ = is the percentage of Trouble Tickets that require intervention by field technicians.
- ${{\%}}_{Call}$ = is the percentage of Trouble Tickets for which a call to the customer by the Contact Center is required.
- ${{\%}}_{Fine}$ = is the percentage of Trouble Tickets for which a fine must be paid to the Retail Operator.
- ${\#}_{Call}$ = is the annual quantity of Trouble Tickets for which a call to the customer by the Contact Center is required.
- ${\#}_{Fine}$ = is the annual amount of Trouble Tickets for which a fine must be paid to the Retail Operator.
- $hours \, per \, day$ = total hours in a day.
- $days \, per \, week$ = total days in a week.
- $weeks \, per \, year$ = total weeks in a year.

Hence, these are the factors mentioned earlier:

- *A1*: Annual cost due to the processing times of the Trouble Tickets processed in a year by the NOC. (Company Resource).
  3 $${\text{A}}1 = {\text{FTE}} * {{\EUR}}_{{{\text{Salary}}}} = \left( {\frac{{\# {\text{TT}} * {\text{t}}_{{{\text{TT}}_{{{\text{NOC}}}} }} }}{{{\text{hours}}\;{\text{per}}\;{\text{day}} * {\text{days}}\;{\text{per}}\;{\text{week}} * {\text{weeks}}\;{\text{per}}\;{\text{year}}}}} \right) * {{\EUR}}_{{{\text{Salary}}}}$$
- *A2*: Annual cost due to the number of Trouble Tickets processed in a year by the NOC (Outsourced Resource). In contracts, this cost is typically paid as a fee for each circuit; this formula can provide an indication of the considerations that the supplier could make on the costs at its own expense.
  4 $${\text{A}}2 = \# {\text{TT}} * {{\EUR}}_{{{\text{TT}}_{{{\text{NOC}}}} }}$$
- *B1*: Annual cost due to the processing times of the Trouble Tickets by the field technicians (Company Resource).
  5 $${\text{B}}1 = {\text{FTE}} * {{\EUR}}_{{{\text{Salary}}}} = \left( {\frac{{\# {\text{TT}} * \%_{{{\text{{{On - Field}}}}}} * {\text{t}}_{{{\text{TT}}_{{{\text{{{On - Field}}}}}} }} }}{{{\text{hours}}\;{\text{per}}\;{\text{day}} * {\text{days}}\;{\text{per}}\;{\text{week}} * {\text{weeks}}\;{\text{per}}\;{\text{year}}}}} \right) * {{\EUR}}_{{{\text{Salary}}}}$$
- *B2*: Annual cost due to the number of Trouble Tickets processed by the field technicians (Outsourced Resource).
  6 $${\text{B}}2 = \# {\text{TT}}_{{{\text{{{On - Field}}}}}} * {{\EUR}}_{{{\text{TT}}_{{{\text{{{On - Field}}}}}} }} = \left( {\# {\text{TT}} * \%_{{{\text{{{On - Field}}}}}} } \right) * {{\EUR}}_{{{\text{TT}}_{{{\text{{{On - Field}}}}}} }}$$
- *C1*: Annual cost due to the processing times of calls to end-users for booking an appointment by the Contact Center (Company Resource).
  7 $${\text{C}}1 = {\text{FTE}} * {{\EUR}}_{{{\text{Salary}}}} = \left( {\frac{{\# {\text{TT}} * \%_{{{\text{Call}}}} * {\text{t}}_{{{\text{TT}}_{{{\text{Call}}}} }} }}{{{\text{hours}}\;{\text{per}}\;{\text{day}} * {\text{days}}\;{\text{per}}\;{\text{week}} * {\text{weeks}}\;{\text{per}}\;{\text{year}}}}} \right) * {{\EUR}}_{{{\text{Salary}}}}$$
- *C2*: Annual cost due to calls to end-users for appointment booking by the Contact Center (Outsourced Resource).
  8 $${\text{C}}2 = \#_{{{\text{Call}}}} * {{\EUR}}_{{{\text{Call}}}} = \left( {\# {\text{TT}} * \%_{{{\text{Call}}}} } \right) * {{\EUR}}_{{{\text{Call}}}}$$
- *D*: Fines to be paid to Retail Operators if the SLA is not respected.
  9 $${\text{D}} = \#_{{{\text{Fine}}}} * {{\EUR}}_{{{\text{Fine}}}} = \left( {\# {\text{TT}} * {{\% }}_{{{\text{Fine}}}} } \right) * {{\EUR}}_{{{\text{Fine}}}}$$

The developed model gives the opportunity to accurately evaluate the $\#TT$ parameter thanks to the data made available by Open Fiber; the parameter is calculated as follows:

10 $$\#TT=\#CB*{\xi }_{{\%}}$$

where:

- $\#CustomerBase (\#CB)$ = amount of end-users making up the Customer Base that the Retail Operators have activated or will activate on the infrastructure of the Wholesale Operator.
- ${\xi }_{{\%}}$ = percentage of customers for which there is an attenuation beyond the threshold established in the SLA. This value is obtained considering the Open Fiber’s distribution of the attenuation of the active circuits.

To describe more effectively, Table 1 correlates the Attenuation Threshold that we intend to use in the SLA and the parameter ${\xi }_{{\%}}$, which instead represents the percentage of customers having an attenuation exceeding that specific Attenuation Threshold. The 100% value in the Attenuation Threshold column refers to the maximum attenuation allowed on the PON FTTH infrastructure considered (in our case, 37.4 dB); beyond the 100% value, the circuit can no longer be considered working. The variables on a network infrastructure can be many, especially since the technologies used can differ between different infrastructures. For this reason, we reserve the right to show the attenuation thresholds (and the maximum acceptable attenuation value) in percentage terms.

**Table 1:** Percentage of customers ${\upxi }_{{\%}}$ who exceed the specific attenuation in the attenuation threshold (%) column.

Attenuation threshold (%)	${\upxi }_{{\%}}$
0	100
5	100
10	100
15	100
20	100
25	100
30	100
35	100
40	100
45	100
50	99.9808
55	99.9425
60	96.4827
65	67.6826
70	33.6161
75	16.6667
80	9.1863
85	5.5779
90	3.7186
95	0.7476
100	0

As described above, the ${\xi }_{{\%}}$ parameter represents the percentage distribution of the attenuation of the active circuits on the PON FTTH network infrastructure of Open Fiber. Here are some examples to facilitate understanding of the table:

- By setting an attenuation threshold equal to 55% of the maximum allowed attenuation on the PON FTTH infrastructure in question, 99.9425% of circuits exceed this attenuation threshold.
- By setting an attenuation threshold equal to 90% of the maximum allowed attenuation on the PON FTTH infrastructure, 3.7186% of lines exceed this attenuation threshold.

**Figure 7:**
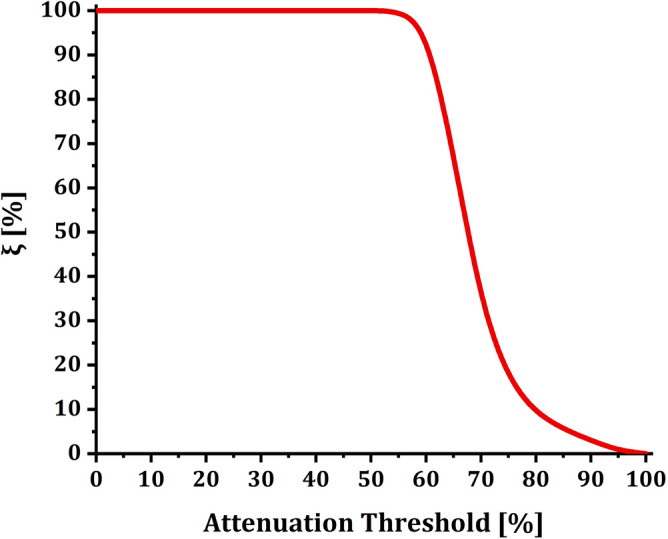
Attenuation Threshold vs ${\xi }_{{\%}}$. The figure shows the percentage of customers ${\upxi }_{{\%}}$ who exceed the specific attenuation indicated in the Attenuation Threshold [%] column.

Table 1 is plotted in Fig. 7 below. Most end-users present an attenuation that falls between 50% and 90% of the maximum acceptable attenuation of the considered PON FTTH infrastructure. No end-user has less than 45% attenuation.

It is now possible to introduce the necessary steps to show the use of the cost model. Furthermore, an example of use is provided with fictitious parameters relating to the case of the Wholesale Operator, who needs to set the SLA for the first time.

The following actions are required to use the cost model:

1. Identify the maximum attenuation the PON FTTH infrastructure under analysis can support without degradation problems. This value will correspond to the $100\mathrm{\%}$ value of the “Attenuation Threshold [%]” column of Table 1. To find this value, refer to the methodology used for this work.
2. Identify the attenuation value (in percentage) at which it is assumed to set the SLA threshold.
3. Calculate the $\#TT$ parameter; this parameter is crucial as it allows you to determine the actual number of customers impacted by the SLA being analyzed; in fact, it is addicted to:
  - Total Customer Base of Retail Operators managed by the Wholesale Operator.
  - Percentage of customers who have a higher attenuation than the chosen threshold.
4. Calculate the formulas that make up the cost model that best fits the context under evaluation.
5. Add up the components of the cost model to get the total cost that would result from setting the attenuation threshold to the previously established value. If the threshold is adequate, report the value in dB; this value will constitute the SLA that will be used on the contracts.

A practical example shows how to use the cost model to complete the discussion—the values used in the example are purely random:

1. After analyzing the degradation on the PON FTTH infrastructure, the maximum attenuation that can be supported by this without incurring degradation events is about 37 dB. This value is equivalent to 100% of the maximum attenuation—shown in the first column of Table 1—that the infrastructure can support.
2. In the contractual SLAs to Retail Operators, it is established to set a threshold of 35 dB to leave a margin of 2 dB with respect to the maximum attenuation allowed by the PON FTTH infrastructure. Therefore, having chosen an acceptable attenuation limit of 35 dB over a maximum attenuation of 37 dB, the threshold is equal to 95% of the maximum attenuation that the infrastructure can support. As we can see from the first column of Table 1, for an attenuation threshold of 95% we have ${\xi }_{\mathrm{\%}}=0.7476\mathrm{\%}$ of customers having an attenuation exceeding this threshold.
3. Assuming that the total number of Retail Operators’ customers who have active service on the Wholesale Operator's infrastructure is equal to 1 million users, it is possible to calculate the $\#TT$ parameter as follows:
  11 $$\# {\text{TT}} = \# {\text{CB}} * \xi_{\% } = 10^{6} * 0.007476 = 7476$$
4. For simplicity, the calculation of only one element of the cost model is reported. For example, the $A1$ parameter introduced above—i.e., annual cost due to the processing times of the Trouble Tickets by the NOC:
  12 $${\text{A}}1 = {\text{FTE}} * {{\EUR}}_{{{\text{Salary}}}} = \left( {\frac{{\# {\text{TT}} * {\text{t}}_{{{\text{TT}}_{{{\text{NOC}}}} }} }}{{{\text{hours}}\;{\text{per}}\;{\text{day}} * {\text{days}}\;{\text{per}}\;{\text{week}} * {\text{weeks}}\;{\text{per}}\;{\text{year}}}}} \right) * {{\EUR}}_{{{\text{Salary}}}} = {{\EUR}} \, 26956,73$$
  where:
  $$\#TT=7476 (as \, previously \, calculated)$$
  $${t}_{{TT}_{NOC}}=0.25 h$$
  $${{\EUR}}_{Salary}= {{\EUR}} 30,000$$
  $$hours \, per,\, day=8$$
  $$days \, per \, week=5$$
  $$weeks \, per \, year=52$$
5. Under these conditions, there would be an annual cost of approximately ${{\EUR}} 26,957$ for the management of Trouble Tickets by the NOC staff (parameter $A1$) for a first diagnosis and a possible remote resolution. As for the other formulas introduced above, they all depend on $\#TT$. The higher the number of customers who exceed the SLA attenuation threshold, the higher the number of Trouble Tickets ($\#TT$). Consequently, very high costs are obtained by adding all the other cost parameters envisaged by the specific context of the Wholesale Operator.

Comparing different thresholds is easily achievable by applying the same procedure with a different threshold and making the appropriate comparisons between the two hypothesized situations. To give an example, by carrying out the same considerations for an attenuation threshold equal to 85% (i.e., a threshold equal to approximately 31.5 dB), we would have ${\xi }_{{\%}}=5.5779$ and, always considering the same number of end-users assumed previously, we get $\# TT =55779$. This result leads to $A1 = {{\EUR}} 201,126.20$. It is possible to see how the appropriate choice of the attenuation threshold to be defined as the contractual SLA between the Retail Operator and the Wholesale Operator leads to enormous savings for the latter.

## Conclusions

The experiments conducted demonstrate how essential it is for Wholesale Operators to correctly assess the limits of their PON FTTH infrastructure for an adequate sizing of the SLAs to be included in the contract with the Retail Operators. In the specific case of the analysis, it has been shown that it is possible to accept an attenuation on the Open Fiber’s PON FTTH infrastructure of about 37 dB without any degradation of the service. In order to evaluate the impact in economic terms on the choice of the attenuation threshold in the SLA, a cost analysis model was proposed with the related considerations on the savings made. The solidity of the cost model is conferred by the distribution of customer attenuations made available by the wholesale operator Open Fiber S.p.A. The consequent collateral benefits of the careful choice of the SLA at issue are equally evident and range on several fronts. As a matter of fact, by lowering the probability of going out of SLA, disputes with Retail Operators are considerably reduced. The Customer Experience of the latter increases since the technicians in the field have more time to dedicate to the activation interventions of their new customers since the lines of those already active have no impact on service quality.

Furthermore, Retail Operators can speed up the troubleshooting processes of their network to identify if there are real causes of degradation not dependent on the Wholesale Operator’s network. Defining an SLA based on a PON FTTH network's actual performance also has environmental sustainability implications. Indeed, a decrease in direct interventions on infrastructures makes it possible to drastically reduce factors such as environmental pollution due to the movement of network teams or the energy used to allow the operational units of the NOC and the Contact Center to process requests. In conclusion, this research aims to offer an experimental analysis for small Wholesale Operators who are now entering the PON FTTH market and those already established internationally to have a point of reference for a conscientious definition of contractual relationships with Retail Operators.

## Supplementary Information

Supplementary Information.

## Data Availability

All data generated or analysed during this study are included in this published article and its Supplementary information files.
